# A Rare Case of Bartter Syndrome Type 3 Presenting With New-Onset Diabetes Mellitus

**DOI:** 10.7759/cureus.76157

**Published:** 2024-12-21

**Authors:** Utkarsh Pradeep, Sourya Acharya, Paschyanti R Kasat, Aman Gupta, Tejas Nehete

**Affiliations:** 1 Department of Medicine, Jawaharlal Nehru Medical College, Datta Meghe Institute of Higher Education and Research, Wardha, IND; 2 Department of Radiodiagnosis, Jawaharlal Nehru Medical College, Datta Meghe Institute of Higher Education and Research, Wardha, IND

**Keywords:** autosomal recessive, bartter syndrome, hypokalemia, hyponatremia, nephrocalcinosis

## Abstract

Bartter syndrome is a rare genetic disorder that often presents in the early phase of life and is caused by mutations in multiple genes encoding the transporters and channels, which are responsible for the reabsorption of various ions in the nephrons. Clinically, it presents with vomiting, failure to thrive, and dehydration. Rare instances of acquired Bartter syndrome have been linked to sarcoidosis, tuberculosis, and autoimmune diseases. Here, we discuss the case of a 52-year-old male patient who presented with complaints of multiple episodes of vomiting and mental obtundation. On further evaluation, he was found to have salt-losing tubulopathy, hypokalemia, and metabolic alkalosis. In the absence of genetic studies, the diagnosis of Bartter syndrome poses a diagnostic challenge in clinical practice.

## Introduction

In 1962, Bartter and his colleagues published the first description of Bartter syndrome, an uncommon autosomal recessive disorder [1]. Any one of the five ion transport proteins in the thick ascending loop of Henle can be mutated to cause Bartter syndrome, which manifests clinically as hypokalemia (low potassium levels), metabolic alkalosis, and salt wasting. One in one million people have Bartter syndrome according to the Framingham Heart Study report [2]. The causes of Bartter syndrome type 1, 2, and 3 are, respectively, defective operation of the Na-K-2Cl cotransporter in the luminal membrane [3], the luminal potassium channel [4], and the basolateral chloride channel [5]. Sensorineural deafness is linked to type 4 Bartter syndrome because both ClC-Ka and ClC-Kb transporters exhibit decreased activity [6]. A minor Bartter phenotype known as type 5 can be caused by defects in the calcium-sensing receptor (CaSR) in the thick ascending limb's basolateral membrane [7]. The first four categories of illnesses are all recessive, while type 5 Bartter syndrome is associated with autosomal dominant hypocalcemia hypercalciuria and is caused by gain-of-function mutations in CaSR [8]. Hypokalemia, hyponatremia, hypocalcemia, hypomagnesemia, hypochloremia with metabolic alkalosis, and decreased urine concentrating ability with increased urinary excretion of prostaglandins and calcium are examples of biochemical disorders. The majority of patients have low or normal blood pressure. Long-term Bartter syndrome is known to be associated with nephrocalcinosis (deposition of calcium salts in the parenchyma of kidneys). Aldosterone and rennin levels in plasma are raised. The symptoms of Bartter syndrome are quite similar to those of long-term loop diuretic consumption. Defects in the transport proteins of the thick ascending limb of the loop of Henle cause the loss of luminal-positive electrical transport potential, which is responsible for the paracellular reabsorption of sodium, calcium, and magnesium. Urine loses calcium, magnesium, sodium, and chloride as a result. Volume contraction-induced loss of salt, chloride, and water triggers the renin-angiotensin-aldosterone (RAA) pathway.

There are five primary genetic problems explained. During the prenatal or neonatal stages, Bartter syndrome is frequently observed. The Bartter syndrome-type phenotype is known to be caused by long-term usage of loop diuretics [9]. A Bartter syndrome-like phenotype has been linked to pulmonary tuberculosis [10] and antitubercular drugs (capreomycin and viomycin). A number of medications, such as aminoglycosides [11,12], colistin [13], and amphotericin B [14], have been shown to cause a Bartter syndrome-like phenotype. Autoimmune diseases including Sjögren's illness [15] and granulomatous diseases like sarcoidosis [16,17] have been linked to acquired Bartter syndrome.

## Case presentation

A 52-year-old male patient presented with complaints of multiple episodes of vomiting over the past 20 days. The vomiting was sudden in onset, non-bilious, non-blood stained, and contained food particles. It was insidious in onset, gradually progressive in nature, and was associated with loss of appetite. The patient also complained of generalized weakness, polydipsia, and muscular cramps over the past few days. There was no history of diabetes mellitus, systemic hypertension, or any other comorbidities.

The patient had no history of alcohol intake, chronic systemic illness, chronic ingestion of any medicine, previous hospitalization, or any other urinary complaints. Physical examination revealed a Glasgow Coma Scale (GCS) score of 15/15, a pulse rate of 80 beats per minute, and blood pressure of 110/70 mmHg in the right arm while in a supine position. The patient was oriented to time, place, and person. Other systemic examinations were within normal limits. Laboratory parameters on presentation are depicted in Table 1.

**Table 1:** Laboratory values of the patient ABG: Arterial blood gas; INR: International normalized ratio; HbA1C: Hemoglobin A1C; PO_2_: Partial pressure of oxygen; PCO_2_: Partial pressure of carbon dioxide; HCO_3_: Bicarbonate

Parameters	Values	Normal range
Hemoglobin	9.5 gm%	13-17 gm%
Total leukocyte count	5500 cells/cu mm	4,000-10,000 cells/cu mm
Total platelet count	2.16 lakh/cu mm	1.5-4.1 lakh/cu mm
Mean corpuscular volume	86.1 fl	83-101 fl
Urea	34 mg/dl	19-43 mg/dl
Creatinine	0.8 mg/dl	0.2-1.3 mg/dl
Serum sodium	110 mm/l	135-145 mm/l
Serum potassium	1.9 mm/l	3.5-5.1 mm/l
INR	1	1-1.3
Alkaline phosphatase	69 U/l	38-126 U/l
Alanine aminotransferases	37 U/l	<50 U/l
Aspartate aminotransferase	45 U/l	17-59 U/l
Albumin	3.8 g/dl	3.5-5 g/dl
Total bilirubin	1.4 mg/dl	0.2-1.3 mg/dl
Magnesium	1.3 mg/dl	1.6-2.2 mg/dl
Phosphorous	2.3 mg/dl	2.5-4.5 mg/dl
Urine creatinine	18.9 mmol/l	3.54-24.6 mmol/l
Urinary sodium	27 mmol/l	15-25 mmol/l
Urinary potassium	7.9 mmol/l	3.5-5 mmol/l
Serum aldosterone	57.4 ng/dl	3.1-35.4 ng/dl
Hba1c	8.1%	<6%
Calcium	6.7mg/dl	8.4-10.2 mg/dl
Fasting blood sugar	275 mg/dl	75-120 mg/dl
Post meal blood sugar	357 mg/dl	<200 mg/dl
pH (ABG)	7.491	7.350-7.450
PCO_2_ (ABG)	37.4 mmHg	35-45 mmHg
PO_2_ (ABG)	89 mmHg	80-100 mmHg
HCO_3_ (ABG)	28.4 mmol/L	18-25 mmol/L

**Figure 1:**
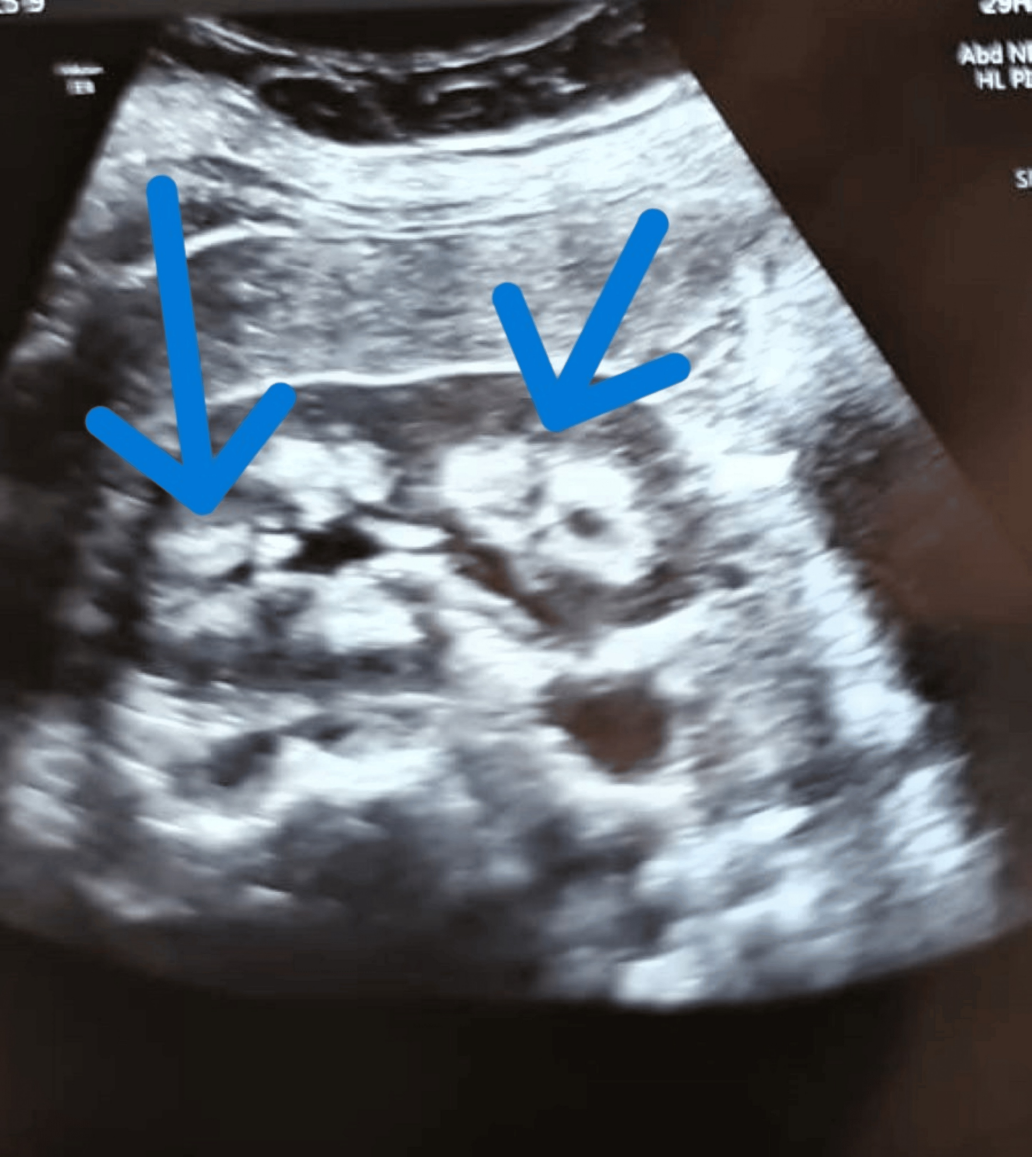
B-mode ultrasonography greyscale image, with arrows indicating medullary nephrocalcinosis in the left kidney of the patient

The patient had severe hypokalemia, which was refractory to persistent potassium corrections that were initially provided. He also had hypocalcemia, hypomagnesemia, and raised hemoglobin A1C (HbA1C) along with elevated fasting and post-meal blood sugar levels. His arterial blood gas analysis was suggestive of metabolic alkalosis. Initially, the patient was started on symptomatic treatment and given intravenous hydration, electrolyte corrections, and injection insulin regular (6 units before breakfast, 6 units before lunch, and 6 units before dinner). However, he was found to be refractory to potassium corrections. Upon urinary investigations, increased urinary potassium and sodium losses were seen. Ultrasonography showed increased echogenicity of the renal medulla, which was suggestive of medullary nephrocalcinosis, as shown in Figure 1.

The patient was started on tab spironolactone 50 mg twice a day, and a rapid improvement was noticed. The patient’s weakness improved along with better glycemic control, and symptoms of polydipsia and polyuria also improved. Laboratory investigations showed significant improvement in electrolyte levels. He was discharged on tab spironolactone, injection insulin regular (4 units before breakfast, 4 units before lunch, and 4 units before dinner), and other supportive medications. Upon follow-up after one month, the patient was doing well, with electrolytes within normal range and glycemic levels well controlled.

## Discussion

Bartter syndrome needs to be differentiated from Gitelman syndrome. In our case, there was increased urinary calcium excretion along with decreased magnesium levels, which are associated with hypokalemia, hyponatremia, and hypocalcemia. The patient had a history of mental obtundation since childhood. He had hypokalemia and poor glycemic control with high urinary potassium losses and metabolic alkalosis. Potassium supplements administered were not able to produce the desired rise in potassium levels. He responded well to spironolactone, and his sodium levels improved to 130 mmol/l while potassium levels rose up to 3 mmol/l. With the improvement in electrolyte levels, the patient's glycemic control improved. Whether Bartter syndrome and diabetes mellitus are causally associated is unknown. We did not identify any relationship with other illnesses, including tuberculosis, sarcoidosis, or autoimmune conditions. He was not on long-term loop diuretics or other suspect medications such as aminoglycoside and cisplatin. He had no history of exposure to heavy metals.

Additionally, upon ultrasonography, nephrocalcinosis was noticed, which was suggestive of long-standing Bartter syndrome. So, a diagnosis of idiopathic acquired Bartter syndrome type 3 with newly diagnosed diabetes mellitus was made. In the literature, very few case reports mention the association of Bartter syndrome with type 2 diabetes. Venkat et al. reported Bartter syndrome patients with high insulin concentrations and islets of Langerhans hyperplasia observed at post-mortem [18]. Another case report [19] reported deterioration of glycemic control after a diagnosis of late-onset Bartter syndrome. Nonetheless, hypokalemia causes a reduction in insulin secretion [6]. Glycemic management is improved in Bartter syndrome patients upon electrolyte correction. There are few long-term outcomes data on Bartter syndrome. Most patients of Bartter syndrome 3 have nephrocalcinosis and hypercalciuria; nonetheless, the frequency of symptomatic urolithiasis in Bartter syndrome seems to be relatively low [20]. It has been documented that Bartter syndrome patients experience nephrotic-range proteinuria [21-23]. Upon doing renal biopsies, widespread glomerular and tubulointerstitial lesions with enlarged glomeruli glomerulosclerosis are frequently observed, as well as focal segmental persistent renal illness [20].

Patients with Bartter syndrome type 1 and 2 may experience a more severe progression of chronic renal disease than patients with Bartter syndrome type 2 and 3, as chronic kidney disease is common in individuals with Bartter syndrome [21,24]. Apart from the molecular defect specifically in Bartter syndrome 4, other risk factors that may lead to chronic kidney injury include nephrocalcinosis, persistent dehydration, progressive proteinuria caused by excessive filtration due to renin-angiotensin system activation, low birth weight or premature birth, and nonsteroidal anti-inflammatory drug (NSAID) treatment. There does not appear to be any relationship between the estimated glomerular filtration rate and serum potassium levels in Bartter syndrome patients [21,25]. Although precise data are limited, some people tend to develop end-stage kidney disease. Although it is often linked to diabetes and hypertension, chronic kidney disease of undetermined etiology (CKDu) has emerged over the past few years, which, upon further investigation, is linked to Bartter syndrome [26].

## Conclusions

Because of interactions with various cotransporters and varying degrees of compensation through alternate pathways, Bartter syndrome is a rare salt-losing tubulopathy caused by many genetic abnormalities and distinct types of mutations along with a poor phenotype-to-genotype association. The identification of the underlying gene mutations has led to some knowledge of Bartter syndrome and highlights the value of genetic testing. Low electrolyte levels and higher excretion in urine were correlated with poor glycemic control in our patient. Diabetes mellitus was the only component found to be linked with Bartter syndrome in our case, which might have been a chance finding.
